# Atypical polyneuropathy, organomegaly, endocrinopathy, monoclonal protein, and skin changes syndrome without polyneuropathy: A case report

**DOI:** 10.1097/MD.0000000000020812

**Published:** 2020-07-02

**Authors:** Qiang Li, Fang Xu, Jing-Feng Duan, Yu-Feng Tang

**Affiliations:** a Department of Neurology; b Department of Hematology, Mianyang Central Hospital, Mianyang, China.

**Keywords:** polyneuropathy, organomegaly, endocrinopathy, monoclonal protein, and skin changes syndrome, polyneuropathy, vascular endothelial growth factor

## Abstract

**Introduction:**

Polyneuropathy, organomegaly, endocrinopathy, monoclonal protein, and skin changes (POEMS) syndrome is a rare paraneoplastic syndrome that occurs secondary to an underlying plasma cell disorder. The diagnosis of POEMS syndrome is 2 of the mandatory major criteria (polyneuropathy and monoclonal plasma cell disorder), 1 of the other major criteria (Castleman's disease, sclerotic bone lesions, or vascular endothelial growth factor elevation), and at least 1 of the minor criteria (organomegaly, extravascular volume overload, endocrinopathy, skin changes, papilledema, thrombocytosis, or polycythemia). However, some cases do not fully meet the diagnostic criteria, such cases are referred to as incomplete or atypical POEMS syndrome.

**Patient concerns:**

A 58-year-old Chinese female was admitted to our department of neurology with weakness of both arms and legs. In addition,it's found that she had skin manifestations, lymphadenopathies, pedal edema, immunoglobin - A-λ restricted paraproteinemia, and elevated vascular endothelial growth factor and other features**, b**ut without polyneuropathy.

**Diagnoses:**

we made a diagnosis of atypical POEMS syndrome without polyneuropathy.

**Interventions:**

Two drug regimens were recommended: VAD (Vincristine, Adriamycin, Dexamethasone) and bortezomib. Finally, the VAD strategy was performed.

**Outcomes:**

The patient's limb strength and pain improved and enzyme parameters decreased gradually after 4 weeks. However, the treatment was still not perfect. Conclusion: We reported a rare case of POEMS syndrome without polyneuropathy. We hope similar cases will be reported in the future.

## 1 Introduction

polyneuropathy, organomegaly, endocrinopathy, monoclonal protein, and skin changes (POEMS) syndrome is a rare paraneoplastic syndrome that occurs secondary to an underlying plasma cell disorder. In previous decades, many cases of POEMS have been reported worldwide. However, the pathogenesis of this condition is still unclear. Many studies have shown that it may be associated with human herpes virus 8 (HHV-8), proinflammatory cytokines, and high expression of vascular endothelial growth factor (VEGF).^[1–3]^ The diagnosis of POEMS syndrome is 2 of the mandatory major criteria (polyneuropathy and monoclonal plasma cell disorder), 1 of the other major criteria (Castleman's disease, sclerotic bone lesions, or VEGF elevation), and at least 1 of the minor criteria (organomegaly, extravascular volume overload, endocrinopathy, skin changes, papilledema, thrombocytosis, or polycythemia). The diagnostic criteria are listed in Table 1. ^[4]^ Among the diagnostic criteria, neuropathy is the dominant characteristic of the syndrome. Among documented cases of POEMS syndrome, all of them have included peripheral nerve damage.^[5]^ However, Ryuji Morizane's team reported a case of atypical POEMS syndrome without polyneuropathy in 2008.^[6]^ Here, we describe the case of a patient who presented with inflammatory myopathy and several typical characteristics of POEMS syndrome, including skin manifestations, lymphadenopathies, pedal edema, IgA-λ restricted paraproteinemia, and elevation of VEGF and other features, but peripheral nerve conduction tests were normal. **T**herefore, we also made a diagnosis of atypical POEMS syndrome without polyneuropathy.

**Table 1:**
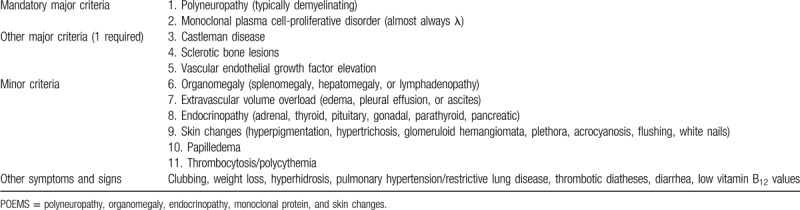
Criteria for the diagnosis of POEMS syndrome.

## 2 Case report

A 58-year-old Chinese female was admitted to our department of neurology with weakness of both arms and legs. Two months prior to admission, she had developed weakness in her bilateral limbs as well as pain in both shoulders. So, she went to a local hospital and reported that she felt the pain ease after receiving traditional Chinese medicine therapy. However, the weakness of her limbs gradually increased. So, she came to our hospital for further diagnosis and treatment. Her past medical history included well-controlled hypertension and diabetes mellitus. Furthermore, she had experienced pyrexia at times in recent years but without the detection of a raised temperature, and she did not seek diagnosis and treatment. She had no history of smoking, alcohol consumption, or recreational drug use. Her family history was unremarkable.

On admission, her height was 158 cm, body weight was 42 kg, body temperature was 38.9°C, pulse was regular at 122 beats/min, respiratory rate was 28 breaths/min, and blood pressure was 170/98 mm Hg. The physical examination revealed that multiple lymphadenopathies were present in the bilateral axillary and the right side of the middle neck triangle, hyperpigmentation in the abdomen, and bilateral pitting pedal edema. The neurological examination showed bilateral upper and lower limb muscle weakness. The upper and lower extremity strength scores, as determined by manual muscle testing, were 3/5 and 4/5, respectively. Tendon reflexes were absent in all extremities, and pathological reflexes were negative. The patient was lucid, and the neurological examination revealed no cranial nerve abnormalities.

**Figure 1:**
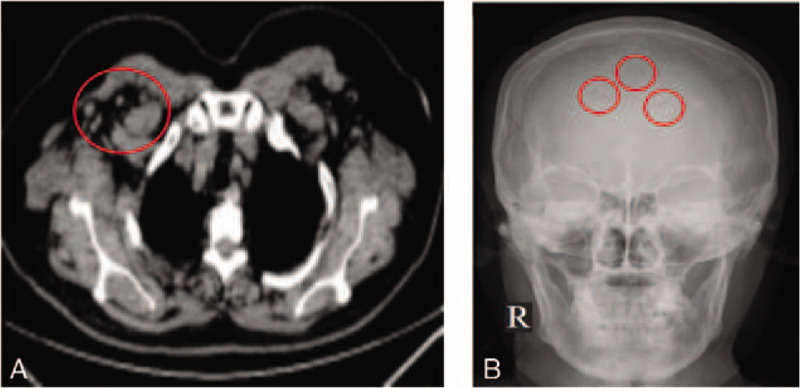
Thorax computed tomography and X-ray of skull.

Laboratory data on admission (Table 2) revealed an elevated platelet count, but the coagulation profile was normal. The erythrocyte sedimentation rate was 41 mm/h (normal range 0 to 20 mm/h). The concentration of C-reactive protein was increased. The testing showed strongly raised creatine kinase (CK) activity (7845 U/L, normal range 40–200 U/L) and elevated liver enzymes in the serum. Blood tests showed a glucose level of 5.88 mmol/L and a glycosylated hemoglobin level of 7.1%. The renal function testing revealed that her creatinine level was normal, but Cystatin C was elevated, and her glomerular filtration rate was decreased. Meanwhile, her urine protein content was 133.35 mg/L (normal range 0 to 10 mg/L). Furthermore, her creatine kinase-MB, MB, and hs-TnT concentrations were significantly increased. Thyroid function tests revealed hypothyroidism. The thyroxin stimulating hormone level was 12.069 uIU/mL (normal range 0.35–4.94 uIU/mL), but the free-T3 and T4 concentrations were normal. The adrenocorticotropic hormone concentration was 3.24 pg/mL (normal range 4.7 to 48.8 pg/mL). The cortisol concentration was 1.1 ug/dL in 4pm (normal range 2.9 to 17.3 ug/dL). Human immunodeficiency virus, serology for hepatitis B and C, and tumor markers (cancer antigen 125, alphafetoprotein, careinoembryonic, carbohydrate antigen 199, cancer antigen 153, cancer antigen 242, prostate specific antigen and neuron specific enolase) were negative. Electrodiagnostic study showed that the motor conduction velocities, sensory nerve action potential, compound muscle action potential, and F wave latency were in the normal range in the bilateral median, ulnar, sural, and tibial nerves. Electromyography analysis showed there were mass abnormal spontaneous activities (fibrillation and positive sharp wave) in the left first dorsal interossei, biceps brachii, tibialis anterior, and the medial head of quadriceps femoris muscle, and myotonic potential in the left iliopsoas and tibialis anterior muscles. The neck triangle lymph node biopsy showed lymphadenosis, but the patient refused an immunohistochemical examination. On the thorax computed tomography (CT) scan, multiple enlarged axillary lymph nodes were observed (Fig. 1A). The flexor carpi radialis muscle biopsy showed many infiltrated lymphocytes, and the congo red staining test was negative. Combined with the symptoms, physical examination, and laboratory tests, we made a diagnosis of myositis. Hence, we treated the patient with methylprednisolone. However, she did not show a significant improvement, despite receiving standard therapy. Thus, immunoelectrophoresis was performed. Immunoelectrophoresis showed monoclonal IgA lambda serum. A bone marrow biopsy examination revealed that the plasma cell content was slightly elevated (0.84%). Furthermore, the concentrations of serum IgA and lambda free light chains were elevated. Serum IgG, IgM, and IgE levels and complements of C3, C4, and kappa free light chains were within the normal range. Furthermore, the VEGF concentration was slightly elevated. In addition, a X-ray of the skull revealed a small bone defect in the frontal lobe (Fig. 1B). Ultrasonography showed no cardiomegaly, hepatosplenomegaly, or pleuroperitoneal fluids. Based on the observed skin manifestations, lymphadenopathies, pedal edema, IgA-λ restricted paraproteinemia, plasma cell dyscrasia in bone marrow, elevation of VEGF, and other features, we made a diagnosis of atypical POEMS syndrome without polyneuropathy.

**Table 2:**
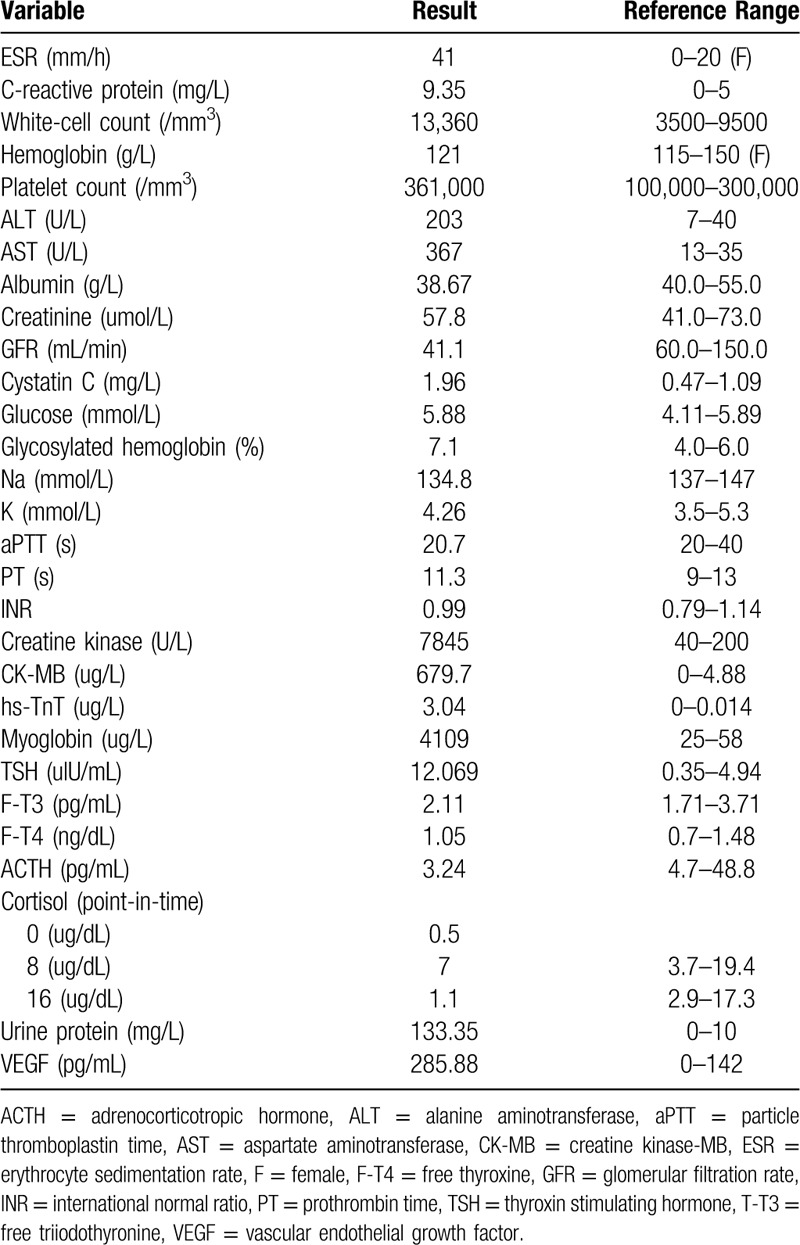
Laboratory data.

## 3 Discussion

At admission, polymyositis was the most probable diagnosis. The patient presented with progressive weakness and raised creatine kinase (CK) and liver enzyme concentrations. The muscle biopsy showed many infiltrated lymphocytes. However, clinical symptoms did not improve after treatment with standard dose of methylprednisolone. Meanwhile, the immunoelectrophoresis assessment showed the monoclonal IgA lambda serum type and a slightly elevated VEGF concentration, and the bone marrow biopsy examination revealed a slightly elevated plasma cell content. So, polymyositis was ruled out. POEMS syndrome became the most likely possibility. In 2000, Goebels reported a case of POEMS syndrome characterized by elevated creatine kinase.^[7]^

POEMS syndrome is a rare paraneoplastic disorder with underlying plasma cell dyscrasia that leads to damage to multiple systems.^[5,8]^ Of the diagnostic criteria released by the Mayo clinic, polyneuropathy is the dominant characteristic of POEMS syndrome.^[4]^ A number of retrospective studies have proven that the prevalence of polyneuropathy is 100%.^[9,10–13]^ However, Ryuji Morizane's team reported a case of atypical POEMS syndrome without polyneuropathy in 2008.^[6]^ A 38-year-old female noticed skin eruptions on her face and torso. In the following years, the patient gradually presented with other several characteristics of POEMS syndrome, including M-proteinemia, sclerotic bone lesions, increased serum VEGF levels, splenomegaly, amenorrhea with hypothalamic gonadal hypofunction, skin changes, and bilateral papilledema, but who remained free of any symptoms of polyneuropathy 5 years after the onset of skin change. Here, we have described a patient with inflammatory myopathy, skin manifestations, lymphadenopathies, pedal edema, IgA-λ restricted paraproteinemia, and elevated VEGF and other features, with normal peripheral nerve conduction tests. So, we diagnosed the case as atypical POEMS syndrome without polyneuropathy.

POEMS syndrome is a treatable disease, but there is no standard therapy for the syndrome. The best therapy choices have been derived from case series. These treatment options include radiation, chemotherapy, hematopoietic stem cell transplantation, and other strategies.^[4]^ The use of plasmapheresis and intravenous immunoglobulins seems invalid.^[14]^ If there is no involvement of the bone marrow, as assessed by iliac crest biopsy, and only 1 to 3 bone lesions, radiation is the first-line therapy. In addition, systemic therapy is the most reasonable treatment option.^[15]^ Chemotherapy drugs used include thalidomide, lenalidomide, alkylating chemotherapy agents, bortezomib, and other drugs.^[4]^ Bevacizumab is a monoclonal antibody that targets VEGF. Some case reports have shown misleading results with bevacizumab and worsening of disease leading to death.^[16,17]^ However, other case studies have shown a benefit, though these have been limited by short or unclear follow up periods, and bevacizumab has been used following, or in addition to, other treatments including cyclophosphamide with dexamethasone.^[18,19]^ At present, high-dose chemotherapy-conditioned autologous stem cell transplantation is the gold standard treatment for POEMS syndrome, showing good hematological control, neurological response, and good survival rates.^[20]^

On admission, we prepared to treat the patient with systemic chemotherapy after autologous stem cell transplantation. Two drug regimens were recommended: VAD (Vincristine, Adriamycin, Dexamethasone) and bortezomib. However, the patient refused autologous stem cell transplantation. Finally, the VAD strategy was performed. The patient's limb strength and pain improved and enzyme parameters decreased gradually after 4 weeks. However, the treatment was still not perfect. We were prepared to give bortezomib in the next phase. Unfortunately, both expense and the possibility of complications precluded the administration of a longer course of systemic therapy.

In conclusion, we have reported a rare case of POEMS syndrome, in which the patient initially presented with weakness of both arms and legs. The patient showed many clinical manifestation of POEMS syndrome, IgA-λ restricted paraproteinemia, plasma cell dyscrasia in the bone marrow, and slightly elevated VEGF, but peripheral nerve injury was absent. So, the diagnosis of the case was atypical POEMS syndrome without polyneuropathy. Up until now, POEMS syndromes without polyneuropathy have rarely been reported. We hope similar cases will be reported in the future to allow definite recommendations to be made.

## Author contributions

The clinical investigation was performed by Qiang Li and Fang

Xu. Qiang Li wrote the case report. Jing-Feng Duan and Yu-Feng Tang wrote the rest of the paper. All the authors agreed to the results and have contributed substantially to the work reported.
